# Extrarespiratory, Digestive, and Hepatic Manifestations of COVID-19 in a Moroccan Series

**DOI:** 10.1155/sci5/3524776

**Published:** 2025-02-26

**Authors:** Hakima Abid, Safae karim, Nada Lahmidani, Wafae Hammoumi, Aicha Attar, Maryame El khayari, Abdelilah Benslimane, Maria Lahlali, Asmae Lamine, Dafr allah Benajah, Sidi Adil Ibrahimi, Mohammed El Abkari, Mohammed El Azami El Idrissi, Ikram Khoussar, Naoual Oubelkacem, Noufissa Alami Drideb, Zineb Khammar, Rhizlane Berrady, Mounia El yousfi, Bahia Bennani

**Affiliations:** ^1^Department of Gastroenterology, Hassan II University Hospital Center, Fez, Morocco; ^2^Department of Fundamental Sciences, Laboratory of Human Pathology Biomedicine and Environment, Faculty of Medicine, Pharmacy and Dentistry of Fez (FMPDF), Sidi Mohammed Ben Abdellah University (USMBA), URL-CNRST No. 15, Fez, Morocco; ^3^Department of Fundamental Sciences, Laboratory of Epidemiology, Faculty of Medicine, Pharmacy and Dentistry of Fez (FMPDF), Sidi Mohammed Ben Abdellah University (USMBA), Fez, Morocco; ^4^Department of Internal Medicine, Hassan II University Hospital Center, Fez, Morocco

**Keywords:** case series, clinical presentation, COVID-19, Morocco, SARS-CoV-2

## Abstract

**Background:** Coronavirus disease 2019 (COVID-19) has emerged as a global human health threat. While SARS-CoV-2 infection exhibits fever and respiratory symptoms, extrarespiratory manifestations were also reported in many cases.

**Objectives:** This study aimed to determine the prevalence of digestive and hepatic symptoms at the onset of infection and to assess whether digestive symptoms are associated with severe disease progression.

**Patients and Methods:** Prospective study was conducted during the first COVID-19 wave (from April to October 2020). It included consenting Moroccan patients diagnosed with COVID-19 based on PCR test and chest computed tomography.

**Results:** A total of 211 patients participated in the study. The patients mean age was 42.3 years, with a sex ratio (F/M) of 1.7. Digestive symptoms were present in 28% of cases, with the most common being nausea or vomiting (12.8%), diarrhea (11.4%), abdominal pain (5.2%), and anorexia (16.6%). These symptoms were significantly associated with diabetes and hypertension. Patients with digestive symptoms reported a significantly higher frequency of anosmia and headache. Hepatic manifestations were present in 21.3%, and digestive symptoms were significantly associated with higher prevalence of liver function disturbances, particularly cholestasis. Nearly half of the patients with digestive symptoms (49.2%) experienced moderate COVID-19, with a higher percentage observed (61.8%) among those aged 42 years or older. However, this association was not statistically significant.

**Conclusion:** Healthcare professionals need to recognize the range of gastrointestinal and hepatic symptoms to ensure timely diagnosis and effective patient management.

## 1. Introduction

Coronavirus disease 2019 (COVID-19) has emerged as a global health concern. While the majority of COVID-19 patients exhibit fever and respiratory symptoms, infection with SARS-CoV-2 can extend beyond the respiratory system, leading to manifestation of extrarespiratory symptoms. Gastrointestinal (GI) involvement is well documented in both animals and humans during coronavirus infections [1]. Bioinformatics analysis has revealed that angiotensin-converting enzyme 2 (ACE2), the receptor for SARS-CoV-2, is highly expressed not only in alveolar type II (AT2) cells of the lung but also in the glandular cells of gastric and duodenal epithelia [2]. Consequently, enterocytes expressing ACE2 can be invaded by SARS-CoV-2, resulting in malabsorption, unbalanced intestinal secretion, and activation of the enteric nervous system, ultimately leading to diarrhea [2]. Additionally, SARS-CoV-2 indirectly damages the digestive system through a cascade of inflammatory responses [3]. SARS-CoV-2 RNA has been detected in blood and stool samples from COVID-19 patients, even after of the virus has been cleared from the upper airways. This suggests that the virus may actively infect and replicate in the GI tract [4]. This study aims to assess the prevalence of digestive and hepatic symptoms during the initial stages of infection and to investigate any potential correlation between these symptoms and the progression to severe forms of the disease.

## 2. Patients and Methods

This study was conducted as part of the project “Multidisciplinary approach for a contribution to improving the prevention and management of COVID-19 in Morocco.” This prospective survey began in April 2020 and was carried out among patients diagnosed with SARS-CoV-2 at the Hassan II University Hospital of Fez.

All participants provided written informed consent for their inclusion in the study. The research was approved by the ethics committee of the University Hospital of Fez, Morocco (approval no. 12/20).

The study included patients diagnosed with COVID-19 through a positive PCR test and/or pulmonary disease confirmed by the thoracic computed tomography (CT) scans. Clinical, biological, and therapeutic data were recorded for all consenting patients on the first day of admission. Patients with incomplete records were excluded from the study. Based on the severity of COVID-19, patients were categorized into three groups: mild COVID-19, moderate COVID-19, or severe COVID-19, according to specific criteria:

• Mild COVID-19: Symptomatic patients meeting the COVID-19 case definition but without evidence of pneumonia or hypoxia.
• Moderate COVID-19: Patients exhibiting clinical signs of pneumonia and corresponding chest imaging manifestations with maintaining SpO2 ≥ 90% on room air without signs of severe pneumonia.
• Severe COVID-19: Patients showing clinical signs of pneumonia along with one of the following: respiratory rate > 30 breaths/minute, severe respiratory distress, or SpO2 < 90% on room air [5].

### 2.1. Statistical Analysis

Statistical analysis was performed using SPSS software (Version 20). Descriptive statistics, including averages and frequencies, were used to describe all variables appropriately. Continuous variables were presented as means ± standard deviation (SD), while categorical variables were expressed as percentages. Correlation between variables was studied using chi-square or Fisher's exact tests. Results with a *p* value ≤ 0.05 were considered statistically significant.

## 3. Results

A total of 211 COVID-19 patients were included in the study with a mean age of 42.3 years (±17.19) and a sex ratio (F/M) of 1.7. Among the participants, 4.7% had been exposed to smoking (active or passive). The most common comorbidities were diabetes (7.6%), hypertension (6.2%), and cardiovascular diseases (2.4%).

The most frequently reported symptoms were fever (35.4%), headache (31.8%), and cough (27.0%). Digestive symptoms were present in 28% of patients, including nausea or vomiting (12.8%), diarrhea (11.4%), abdominal pain (5.2%), and anorexia (16.6%).

**Figure 1:**
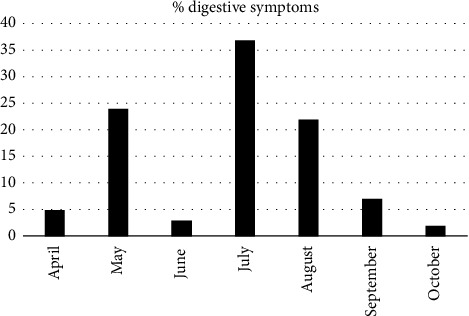
Monthly variations in the percentage of reported digestive symptoms in 2020.

An examination of the reported digestive symptoms across the months during which patients were recruited showed notable variations. Specifically, there was a notable peak of digestive symptoms (37%) noted in July 2020, followed by a sharp decrease to 2% in October 2020 (Figure 1).

Hepatic manifestations were observed in 21.3% of patients, with hepatic cytolysis being the most frequent (62.2%), followed by cholestasis (24.3%) and a combination of both (13.5%). Gamma-glutamyl transferase (GGT) elevation was noted in 35.1% of cases, while alkaline phosphatase (ALP) elevation was observed in 5.4% of cases. Anemia was noticed in 18.7% of cases, lymphopenia in 8.7%, thrombocytopenia in 8.0%, and elevated CRP levels in 33.1% of cases.

Patients were grouped according to COVID-19 severity, 35.2% of patients were with mild disease, 55.1% had moderate disease, and 9.7% had severe disease.

Correlation of extradigestive symptoms with sociodemographic and clinical factors shows that older patients and women were the predominant groups among patients with digestive symptoms. Significant associations were observed between digestive symptoms and comorbidities like diabetes and hypertension. Furthermore, anosmia and headache were notably more common in patients with digestive symptoms, occurring in 55.9% and 54.2% of cases, respectively (Table 1). Hepatic manifestations were also more frequent in this group (35.6%) particularly cholestasis (50.0%) (Table 1). Likewise, severe COVID-19 was observed in 11.9% of patients with digestive symptoms compared to 8.8% of the others, but the association was not statistically significant (Table 1).

Correlation of COVID-19 severity with digestive symptoms according to patients' age groups (< 42 years vs. ≥ 42 years) has been studied. The result shows that moderate COVID-19 severity was more prevalent in patients aged 42 or older with digestive symptoms (61.8%). In contrast, more than half (56.5%) of those under 42 had mild COVID-19. These differences did not reach statistical significance (Table 2).

Regarding treatment, 73.45% of patients received a combination of chloroquine/hydroxychloroquine and azithromycin. During follow-up, digestive side effects were recorded in 41.2%, primarily diarrhea (37.9%), vomiting (6.6%), and abdominal pain (7.1%). No severe effects that require discontinuation of the treatment and no cases of jaundice or pancreatitis were reported or observed in these cases.

A total of 11.84% of patients required intensive care, and the mortality rate was 1.89%. Recovery, characterized by clinical improvement and negative RT-PCR results, occurred in 88.1% of cases.

## 4. Discussion

As of March 27, 2021, approximately 126 million people worldwide had been diagnosed with COVID-19 and 2.77 million had died. The digestive system has emerged not only as a site of disease manifestation but also as a potential factor influencing disease severity and viral transmission. This study is the first one to be conducted in the Fez region of Morocco that aims to determine the prevalence of digestive and hepatic symptoms during the early stages of COVID-19 infection and to explore their potential association with progression to severe forms of the disease.

The prevalence of GI symptoms in our study was 28%, which is comparable to the rates reported in studies by Al Argan et al. (28.5%) [6] and Leal et al. (29.9%) [7], but lower than those reported in China [8, 9]. Among GI symptoms, anorexia was the most commonly reported symptom in 16.6% of cases, followed by nausea/vomiting, diarrhea, and abdominal pain presented in 12.8%, 11.4%, and 5.2% of cases, respectively. A similar distribution was reported in the Chinese study [8], with 39.9%, 13.7%, 10.1%, and 2.2% of anorexia, nausea/vomiting, diarrhea, and abdominal pain cases, respectively. However, diarrhea and nausea/vomiting were the most frequent complaints, reported in other studies [6, 7, 10, 11].

Our study reveals significant fluctuations in the rate of reported digestive symptoms among COVID-19 patients, with a peak of 37% in July 2020 and a decrease to 2% in October 2020. These variations could be influenced by factors such as seasonal changes, which have been shown to affect symptom prevalence, as noted in Iranian study [12]. Additionally, different COVID-19 variants exhibit distinct symptom profiles, which might contribute to these observed fluctuations [13]. However, in Morocco during the first wave of COVID-19, which began in March 2020, the virus was predominantly associated with the original Wuhan strain and its early mutations [14]. Therefore, the association of symptoms with specific COVID-19 variants cannot be conclusively established. Furthermore, it is important to note that changes in public health measures could impact the symptom reporting and recording, as highlighted in Moynihan et al.'s review [15].

The results of our study indicate that a higher percentage of patients with digestive symptoms are over 42 years old. This finding aligns with previous research suggesting that older adults are more likely to experience severe or additional symptoms, including GI issues, during COVID-19 [16, 17]. Moreover, the predominance of female patients with digestive symptoms (62.7%) is consistent with results reported in other geographical regions [18, 19] and may be attributed to gender differences in immune response or hormonal influences.

In our study, it is important to point out that the prevalence of severe COVID-19 was more common among patients with GI symptoms (11.9%) compared to the others (8.8%). However, the association is not statistically significant. This finding aligns with the results reported by Kaiyasah et al., suggesting that the presence of GI symptoms does not influence the severity of pneumonia in COVID-19 patients [20]. Similarly, Shousha et al. found that the majority of cases with digestive symptoms (83.4%) experienced non-severe forms of COVID-19 [21]. In contrast, other studies reported a significant association between GI symptoms and severe COVID-19 [22, 23]. This discrepancy may be related to the limited sample size (in our study) and/or to variations in viral load in the GI tract, or to eventual ethnic difference of ACE2 expression rate in GI cells [4]. Moreover, the predominance of COVID-19 severe cases among old patients compared to young ones is consistent with results reported by several studies [24–26]. The association between advanced age and increased COVID-19 severity may be attributed to the age-related decline in cell-mediated immunity and a reduced humoral immune response.

Abnormal liver function has been reported in approximately 15%–50% of COVID-19 patients in China [27, 28]. However, the underlying mechanisms of liver damage in these patients remain unclear. It has been suggested that virus-induced cytopathic effects may directly contribute to liver injury. In another study, a postmortem liver biopsy obtained from a COVID-19 patient shows moderate microvesicular steatosis and mild lobular and portal activity, consistent with either direct viral infection or drug-induced liver injury; however, hepatocyte viral inclusions were not identified [29]. In fact, data from two independent cohorts revealed a significant enrichment of ACE2 expression in cholangiocytes (59.7% of cells) compared to hepatocytes (2.6% of cells) suggesting that SARS-CoV-2 could bind directly to ACE2-positive cholangiocytes to deregulate liver function [30]. In our study, hepatic manifestations were present in 21.3% of cases with a predominance of hepatic cytolysis (62.2% of patients with hepatic manifestation), cholestasis was noted in 24.3% of cases, and both hepatic cytolysis and cholestasis were observed in 13.5% of patients. GGT and ALP were elevated in 35.1% and 5.4% of cases, respectively.

However, a Chinese study found that 54% (30/56) of COVID-19 patients had elevated GGT levels, while only 1.8% (1/56) had elevated ALP level [9]. This difference can be explained by several factors: (i) immune-mediated inflammation, which may contribute to liver damage in critically ill COVID-19 patients [31], and (ii) high levels of positive expiratory pressure, which can lead to liver congestion by increasing right atrial pressure and preventing venous return. However, published data suggest that non-mechanically ventilated COVID-19 patients admitted to hospital do not typically show liver abnormalities. It is also important to consider that antiviral drugs (lopinavir/ritonavir), antipyretics (paracetamol), antibiotics (including macrolides and quinolones), and steroids can also induce liver damage [32].

A Chinese study has reported an increase in serum pancreatic enzymes in 17% of COVID-19 patients in Wuhan [33]. This increase was not associated with severe pancreatitis, but the authors noted an acute rise in blood sugar levels, which could suggest elevated ACE2 receptor expression in pancreatic islets cells. In our series, digestive symptoms were significantly more prevalent in diabetic patients (*p*=0.041). Furthermore, a Chinese study demonstrated that diabetes can influence clinical symptoms and the progression of COVID-19 [34]. This effect may be attributed to the presence of autonomic neuropathy and abnormal GI hormone secretion in diabetic individuals, which can contribute to stress-induced GI dysfunction.

This study has some limitations. In fact, patients' data were gathered only at the point of their first COVID-19 infection and during their initial hospital stay. As a result, our analysis relies exclusively on data collected at these initial stages, limiting our ability to evaluate changes over time or track the progression of digestive symptoms. Furthermore, the data were collected during the first wave of the pandemic marked by the Wuhan strain infection. Since then, new SARS-CoV-2 variants have emerged which may have an influence on the associated clinical presentations. This limitation indicates that additional studies are needed to assess the eventual changes in extrarespiratory clinical picture. Thus, the differences in geographical context, environmental factors, lifestyle habits, and genetic factors may also have an influence on the occurrence of extrarespiratory symptoms, an aspect that should not be overlooked.

## 5. Conclusion

Digestive symptoms were frequently observed in patients with COVID-19. However, these manifestations were predominantly of moderate severity, and their presence did not correlate with the progression to severe disease. Nevertheless, healthcare professionals should be familiar with the diverse GI and hepatic symptoms associated with COVID-19 to ensure timely diagnosis and appropriate patient management. Further research studies are needed to explore the relationship between GI manifestations, the presence of SARS-CoV-2 in stool samples, and their impact on the progression of the disease.

## Figures and Tables

**Table 1:** Sociodemographics and clinical characteristics of COVID-19 patients.

	Digestive symptoms, *n* (%)		
	Absence	Presence	*p* value
Age (years)			
< 42	70 (51.9)	23 (40.4)	0.145
≥ 42	65 (48.1)	34 (59.6)	
Gender			
Men	57 (37.5)	22 (37.3)	0.977
Women	95 (62.5)	37 (62.7)	
Passive smoking	55 (27.4)	54 (40.0)	0.385
Comorbidities			
Hypertension	6 (3.9)	7 (11.9)	0.032
Diabetes	8 (5.3)	8 (13.6)	0.041
Cardiovascular disease	3 (2.0)	2 (3.4)	0.544
Symptoms			
Runny rose	24 (15.8)	8 (13.6)	0.685
Dyspnea	15 (9.9)	9 (15.3)	0.269
Sneeze	29 (19.1)	10 (16.9)	0.721
Cough	41 (27.0)	16 (27.1)	0.983
Vertigo	18 (11.8)	5 (8.5)	0.481
Fever	59 (38.8)	16 (27.1)	0.111
Headache	35 (23.0)	32 (54.2)	∼0.001
Anosmia	38 (25.0)	33 (55.9)	∼0.001
Hepatic manifestations	16 (13.9)	21 (35.6)	0.001
Cholestasis	5 (16.7)	9 (50.0)	0.014
Cytolysis	13 (13.7)	15 (26.3)	0.052
COVID-19 severity			
Mild	46 (33.6)	23 (39.0)	0.523
Moderate	79 (57.7)	29 (49.2)	
Severe	12 (8.8)	7 (11.9)	

**Table 2:** Correlation of COVID-19 severity with the presence of digestive symptoms according to age.

COVID-19 severity	Digestive symptoms, *n/N* (%)			
	< 42 years		≥ 42 years	
	Absence	Presence	Absence	Presence
Mild	25/61 (41.0)	13/23 (56.5)	18/60 (30.0)	9/34 (26.5)
Moderate	29/61 (47.5)	7/23 (30.4)	38/60 (63.3)	21/34 (61.8)
Severe	7/61 (11.5)	3/23 (13.0)	4/60 (6.7)	4/34 (11.8)
*p* value	0.356		0.682	

## Data Availability

The datasets generated and/or analyzed during the current study are available from the corresponding author on reasonable request.
